# Optoelectronic Properties of In_0.87_Ga_0.13_As_0.25_P_0.75_(001)β_2_(2×4) Surface: A First-Principles Study

**DOI:** 10.3390/ma16072834

**Published:** 2023-04-02

**Authors:** Yong Wang, Jianxin Li, Junju Zhang, Weiwei Sha

**Affiliations:** Institute of Electronic Engineering and Optoelectronic Technology, Nanjing University of Science and Technology, Nanjing 210094, China; 217104010121@njust.edu.cn (Y.W.);

**Keywords:** In_0.87_Ga_0.13_As_0.25_P_0.75_(001)β_2_(2×4) surface, electronic structure, mulliken population, optical properties, work function

## Abstract

InGaAsP photocathode surface affects the absorption, transport and escape of photons, and has a great influence on quantum efficiency. In order to study InGaAsP photocathode surface, the electronic structure, work function, formation energy, Mulliken population and optical properties of In_0.87_Ga_0.13_As_0.25_P_0.75_(001)β_2_(2×4) reconstruction surface were calculated from first principles. Results show that stabilized the In_0.87_Ga_0.13_As_0.25_P_0.75_(001)β_2_(2×4) surface is conducive to the escape of low-energy photoelectrons. The narrow bandgap and emerging energy levels of the reconstruction surface make the electron transition easier. Under the action of the dipole moment, the electrons transfer from inner layers to the surface during the surface formation process. By contrast to the bulk, the surface absorption coefficient and reflectivity considerably decrease, and the high-reflection range becomes narrower as the falling edge redshifts. On the contrary, the surface transmissivity increases, which is conducive for the photons passing through the surface into the bulk to excite more photoelectrons. Meanwhile, the higher absorption coefficient of surface in low-energy side is favorable for long-wave absorption. The dielectric function peaks of the surface move toward the low-energy side and peak values decrease.

## 1. Introduction

In night vision field, ternary and quaternary III-V alloy semiconductors such as the photoemissive layer can prolong the long-wave threshold of the negative electron affinity (NEA) GaAs photocathodes by adjusting the bandgap, and can work at 1.06 μm wavelength or longer wavelengths [1,2,3,4,5]. At 1.06 μm, Fisher et al. obtained an InGaAs photocathode with 3% quantum efficiency in the laboratory by optimizing the experimental conditions, while Escher et al. obtained an InGaAsP photocathode with quantum efficiency up to 9% [6,7,8]. Thus, quaternary InGaAsP is better for the 1.06 μm wavelength detection system. However, for near-infrared narrow bandgap, InGaAsP photocathodes of cut-off wavelength exceeding 1.1 μm need to overcome a surface barrier that is higher than the vacuum level. Although using bias voltage to form a field-assisted photocathode can effectively solve this problem, Williams and Fisher believe that the work function of Cs_2_O can be reduced to 0.7 eV [9], which means that if the bandgap is greater than 0.7 eV, the photoelectric emission is mainly determined by the bandgap. Based on this judgment, reducing the bandgap and improving the activation technique were attempted to expand the near-infrared wavelength response. Although some achievements have been achieved in the laboratory, overall progress is limited. It is worth noting that the surface properties of InGaAsP photocathode significantly influence its quantum efficiency. Since the atoms on the photocathode surface lack adjacent atoms, the balance of forces between atoms in the three-dimensional structure is broken, which leads to surface relaxation and reconstruction. The properties of the reconstructed surface are different from those of the bulk, and they determine the Cs/O adsorption site and activation method. InGaAsP and GaAs both have zinc-blende structures. There are α, γ, β and β2 reconstruction phases on the As-rich GaAs(001)(2×4) surface [10,11,12,13], and the β_2_(2×4) phase proposed by Chadi has been proven to be the most stable structure when the As coverage is 0.75 mL [14,15]. Thus, the β_2_(2×4) phase is chosen for studying InGaAsP(001) surface.

We constructed As-terminated In_0.87_Ga_0.13_As_0.25_P_0.75_(001)β_2_(2×4) surface models with different atomic configurations. First-principles methods [16] based on the density-functional theory (DFT) [17,18] are used to calculate their electronic structure, work function, surface energy, Mulliken population and optical properties. The average calculation values are taken as the results to ensure the accuracy. Results are analyzed and compared to bulk In_0.87_Ga_0.13_As_0.25_P_0.75_. The work elucidates the In_0.87_Ga_0.13_As_0.25_P_0.75_(001)β_2_(2×4) surface through simulations, and it is instructive for the activation technology of In_x_Ga_1−x_As_y_P_1−y_ photocathodes.

## 2. Computational Details

**Figure 1:**
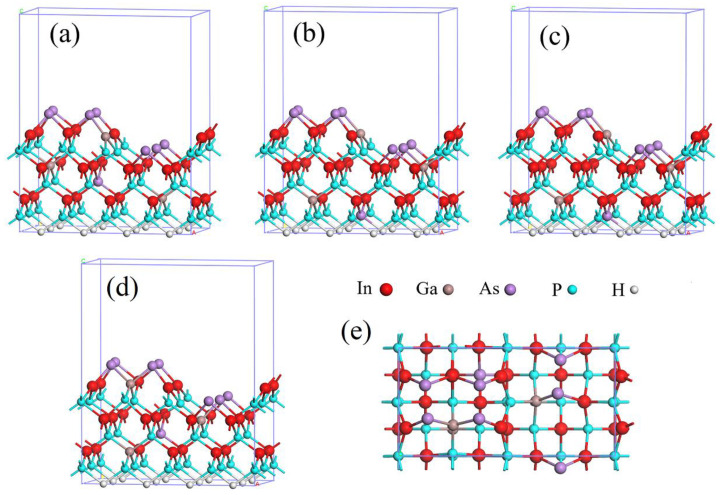
(**a**–**d**) Side views of different atomic configurations of In_0.87_Ga_0.13_As_0.25_P_0.75_(001)β_2_(2×4) surface model, (**e**) top view of (**d**).

For constructing the In_0.87_Ga_0.13_As_0.25_P_0.75_(001)β_2_(2×4) surface model, we first cleave the In_0.87_Ga_0.13_As_0.25_P_0.75_ conventional cell to obtain its (001) surface, and then modify the atoms on the (001) surface to build the β_2_(2×4) phase. The constructed surface model is a slab model with 8 layers of atoms and comprises 7 As atoms, 3 Ga atoms, 21 P atoms, 19 In atoms and 8 H atoms which are used to saturate the bottom dangling bonds. To simulate bulk conditions and real surface, the bottom and top four layers of atoms are, respectively, relaxed and fixed, and a vacuum layer with thickness of 1.5 nm above the surface is built to separate the repeated slabs to prevent them from interacting. Considering the randomness of atomic arrangement in the crystal surface, different atom configurations of surface models are considered in calculation. Meanwhile, atoms are evenly distributed in surface layers as far as possible. In Figure 1, four atom configurations of In_0.87_Ga_0.13_As_0.25_P_0.75_(001)β_2_(2×4) surface model are listed and their average calculated values are adopted to improve the accuracy of result analysis.

The Cambridge Serial Total Energy Package (CASTEP), which is based on DFT, is used in our calculation. Generalized gradient approximation (GGA) [19] along with the Broyden-Fletcher-Goldfarb-Shannon (BFGS) algorithm is adopted to optimize the structure of surface models, and calculation parameters are considered as follows: cut-off energy for the plane wave 420 eV, convergence precision 1 × 10^−6^ eV/atom, monatomic energy converges to below 5 × 10^−6^ eV/atom, maximum displacement ≤ 0.0005 nm and force ≤ 0.001 eV/nm. In the first Brillouin zone [20], the sample value of k points is set as 4 × 6 × 1. Additionally, the valence electrons In:4d^10^5s^2^5p^1^, Ga:3d^10^4s^2^4p^1^, As:4s^2^4p^3^ and P:3s^2^3p^3^ are used in the calculation.

## 3. Results and Discussion

### 3.1. Surface Energy

**Figure 2:**
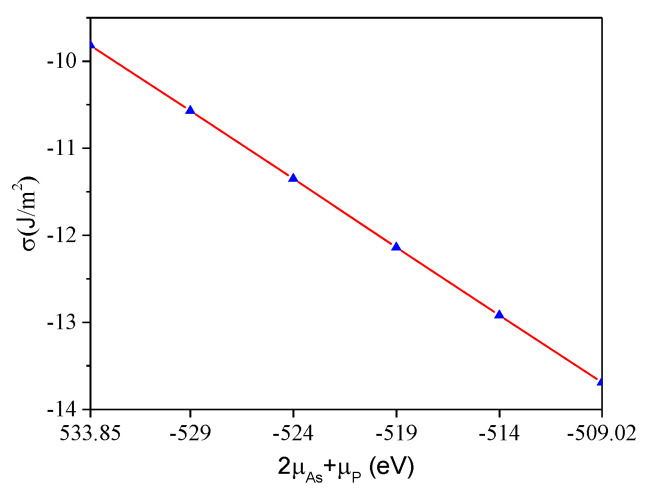
In_0.87_Ga_0.13_As_0.25_P_0.75_(001)β_2_(2×4) surface energy.

The unsaturated bonds appear at the outermost layer of the surface as the lattice terminates here, causing surface reconstruction. The surface energy reflects the stability of the reconstructed surface and it is defined as follows [21]:

(1) $$\sigma =\left(E_{\mathrm{slab}}-{nE}_{\mathrm{bulk}}\right)/A$$

where *E*_slab_ is the slab model energy, *E*_bulk_ and *n* are, respectively, the energy and quantity of the bulk In_0.87_Ga_0.13_As_0.25_P_0.75_ primitive cell, and *A* represents the surface model area. Taking the pseudo-hydrogen atoms into account, the calculation of the surface energy is revised as follows:

(2) $$\begin{array}{ll} \sigma \;= & \left(E_{\mathrm{slab}}-n_{\mathrm{In}}\mu_{\mathrm{In}}-n_{\mathrm{Ga}}\mu_{\mathrm{Ga}}{-n_{P}\mu_{P}-n}_{\mathrm{As}}\mu_{\mathrm{As}}-n_{H}\mu_{H}\right)/A\\ & \approx \left[E_{\mathrm{slab}}-22\times \left(0.87\mu_{\mathrm{In}}+0.13\mu_{\mathrm{Ga}}+0.75\mu_{P}+0.25\mu_{\mathrm{As}}\right)-2\mu_{P}-4\mu_{\mathrm{As}}-8\mu_{H}\right]/A,\\ & =\;\;\;\left[E_{\mathrm{slab}}-\frac{22}{32}\times E_{\mathrm{bulk}}-2\mu_{P}-4\mu_{\mathrm{As}}-8\mu_{H}\right]/A \end{array}$$

where *n*_i_ and *μ_i_* are, respectively, the number and chemical potential of *i* kind atom which involves In, Ga, As, P and H. Here, *μ*_H_ is approximately −12.46 eV. To ensure that the calculated surface is stable, Equation (2) must meet the following requirements:

(3) $$\left\{ \begin{array}{l} {E_{\mathrm{As}}-\left|\mu_{\mathrm{InGaAsP}}\right|<\mu }_{\mathrm{As}}<E_{\mathrm{As}}\\ {E_{P}-\left|\mu_{\mathrm{InGaAsP}}\right|<\mu }_{P}<E_{P}\\ \mu_{\mathrm{InGaAsP}}=E_{\mathrm{bulk}}-0.87E_{\mathrm{In}}-0.13E_{\mathrm{Ga}}-0.75E_{P}-0.25E_{\mathrm{As}} \end{array}\right.$$

where *E*_As_ and *E*_P_ are, respectively, the average chemical potential of As and P atoms in the simple substance phase. As the function of $\mu_{P}+2\mu_{\mathrm{As}}$, the calculated surface energy of In_0.87_Ga_0.13_As_0.25_P_0.75_(001)β_2_(2×4) is plotted in Figure 2. The values of surface energy on line are all negative, indicating that the surface is stable.

### 3.2. Work Function

Surface reconstruction changes the surface conditions and influences the electron escape. Since the work function varies with the surface condition, the work function can be used as an important parameter to characterize whether the photocathode surface easily emits photoelectrons, and it is the lowest energy required for electrons to escape to the vacuum, that is, the energy difference between the vacuum level and the Fermi level, which is as follows [22]:

(4) $$\phi =E_{vac}-E_{f}$$

where *E_f_* and *E_vac_* represent the Fermi and vacuum levels, respectively. In our calculation, the work function of the In_0.87_Ga_0.13_As_0.25_P_0.75_(001)β_2_(2×4) surface is 4.712 eV. The calculated work function of GaAs(001)β_2_(2×4) surface is 4.838 eV [23] lower than its ionization energy 5.5 eV [24]. Compared to the GaAs surface, the work function of In_0.87_Ga_0.13_As_0.25_P_0.75_ surface is smaller. After Cs/O activation, the work function of the In_0.87_Ga_0.13_As_0.25_P_0.75_(001)β_2_(2×4) surface is further reduced, decreasing the energy required for the bulk electrons to be emitted into the vacuum, which extends the response wavelength of In_0.87_Ga_0.13_As_0.25_P_0.75_ photocathode and increases its photoemission efficiency in the near-infrared region.

### 3.3. Electronic Structure

**Figure 3:**
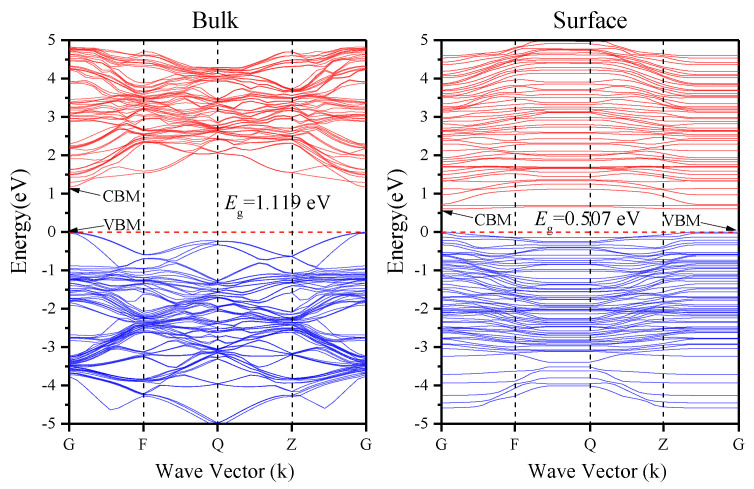
Band structure of bulk In_0.87_Ga_0.13_As_0.25_P_0.75_ and In_0.87_Ga_0.13_As_0.25_P_0.75_ (001)β _2_(2×4) surface.

The band structure of In_0.87_Ga_0.13_As_0.25_P_0.75_ bulk and reconstruction surface are shown in Figure 3, wherein the dashed lines denote the Fermi levels. The calculated bandgap values for the bulk and reconstruction surface are, respectively, 1.119 and 0.507 eV lower than the theoretical values. This is a universal phenomenon caused by the DFT underestimating the bandgap [25]. In_0.87_Ga_0.13_As_0.25_P_0.75_ surface has a narrower bandgap than the bulk, and the conduction band minimum and valence band maximum both appear at the G point, showing that it has direct bandgap, which is conducive to photoelectron excitation. Moreover, the generation of some new energy levels widens the surface energy band, which means the effective electron mass decreases, facilitating the electron diffusion in the surface.

**Figure 4:**
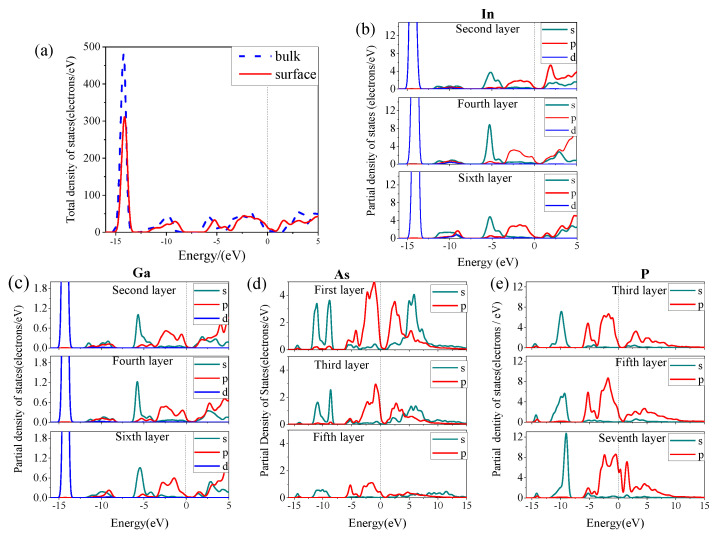
(**a**) Total DOS of the In_0.87_Ga_0.13_As_0.25_P_0.75_ bulk and surface, (**b**–**e**) the partial DOS of the In, Ga, As and P atom in every layer.

The influence of surface reconstruction on the energy bands and electron structure can be further analyzed by the density of states (DOS). The DOS of the In_0.87_Ga_0.13_As_0.25_P_0.75_ bulk and surface are shown in Figure 4, wherein Fermi levels are represented by dotted lines. Compared to the In_0.87_Ga_0.13_As_0.25_P_0.75_ bulk, the total DOS of the surface slightly converges toward the Fermi level, and a new electronic state density peak composed of P 3p, As 4p and In 5p states appears in the range of 0.5–2.2 eV. These changes in the DOS are consistent with the band structure. There are seven layers in the surface model, as shown in Figure 1. We can see from Figure 4b–e that In, Ga, As and P atoms near the top layer contribute more to the electronic states around the Fermi level than atoms in other layers, particularly the As atoms in the first layer, which contribute the most to surface reconstruction.

After reconstruction, the integral partial DOS of the surface considerably changes relative to the bulk. The variation is shown in Table 1 in which the symbols “+” and “–” represent increase and reduce, respectively. Results show that almost all state electrons are reduced except P 3s state electrons. This is mainly due to the appearance of a large number of sp^3^ hybrid orbits during the surface formation process, which neutralizes the dipole moment and stabilizes the surface.

### 3.4. Mulliken Population

In_0.87_Ga_0.13_As_0.25_P_0.75_(001)β_2_(2×4) surface is a polar surface with dipole moment perpendicular to it due to the opposite electronegativity between In (Ga) and As (P) in alternating layers, and belongs to the type 3 surface in Tasker theory [26]. To stabilize the surface, the dipole moment should be canceled through the charge redistribution on In, Ga, As and P atomic orbits. After surface reconstruction, the mean Mulliken charge distribution in each layer of In_0.87_Ga_0.13_As_0.25_P_0.75_(001)β_2_(2×4) surface is presented in Table 2. The positive charges of In and Ga atoms decrease, and the negative charges of As and P atoms significantly increase in the layers nearest to the top. This indicates that electrons transfer from the inner layers to the surface under the action of dipole moment in the surface formation process. Simultaneously, the lengths of In-As, Ga-As, In-P and Ga-P bonds increase, decreasing the polarity and canceling the dipole moment. Consequently, the reconstructed surface stabilizes, and the electron diffusion length from bulk to surface increases.

The charge-transfer index proposed can be used to measure the degree of deviation from the ideal ionic model. The calculation of the charge-transfer index is as follows:

(5) $$c=\frac{1}{N}\sum_{\Omega =1}^{N}\frac{\zeta (\Omega )}{OS(\Omega )}=\langle \frac{\zeta (\Omega )}{OS(\Omega )}\rangle$$

where *N* represents the atom number in the crystal, and OS (Ω) and ζ (Ω) are, respectively, the nominal oxidation states and the topological charge.

According to the description of Mori-Sánchez et al. in their study, the charge-transfer index of most III–V polar compounds is in the range of 0.3–0.6 [27]. We obtained that the charge-transfer index of In_0.87_Ga_0.13_As_0.25_P_0.75_ is 0.43, indicating that our calculation is reliable. Due to H atoms having a low transfer index in the surface bottom, the transfer index of In_0.87_Ga_0.13_As_0.25_P_0.75_(001)β_2_(2×4) surface is decreased to 0.375, smaller than In_0.87_Ga_0.13_As_0.25_P_0.75_ bulk, causing the ionicity of In_0.87_Ga_0.13_As_0.25_P_0.75_(001)β_2_(2×4) surface to become stronger.

### 3.5. Optical Properties

The optical parameters of the In_0.87_Ga_0.13_As_0.25_P_0.75_(001)β_2_(2×4) surface such as dielectric function, absorption coefficient and reflectivity are closely related to the performance of photocathodes. The dielectric function links the band structure to the spectra. Additionally, optical absorption is the first step of the three-step model concerning the photoemission theory of photocathodes proposed by Spicer, which governs the photoelectron excitation. The absorption curve edges determine the working waveband range of photocathodes. These parameters are mainly determined by the electronic structure and the carrier density around the Fermi level in crystal. 

Complex dielectric function can be well described in the linear response range as follows:

(6) $$\varepsilon \left(\omega \right)=\varepsilon_{1}\left(\omega \right)+i\varepsilon_{2}\left(\omega \right)$$

where *ω* denotes the angular frequency, and *ε*_1_ and *ε*_2_ denote the real and imaginary parts of the dielectric function, respectively. On the basis of Kramers–Kronig dispersion relations and the definition of the direct transition probabilities, *ε*_1_ and *ε*_2_ can be expressed as follows [28]:

(7) $$\varepsilon_{1}\left(\omega \right)=1+\frac{2e}{\varepsilon_{0}m^{2}}\cdot \sum_{V,C}\int_{BZ}\frac{2dK}{{\left(2\pi \right)}^{2}}\frac{{\left|a\cdot M_{V,C}\right|}^{2}}{\left[E_{C}\left(K\right)-E_{V}\left(K\right)\right]/\hbar }\cdot \frac{1}{{\left[E_{C}\left(K\right)-E_{V}\left(K\right)\right]}^{2}/\hbar^{2}-\omega^{2}}$$

(8) $$\varepsilon_{2}\left(\omega \right)=\frac{\pi }{\varepsilon_{0}}{\left(\frac{e}{m\omega }\right)}^{2}\cdot \left\{\sum_{V,C}\int_{BZ}\frac{2dK}{{\left(2\pi \right)}^{2}}{\left|a\cdot M_{V,C}\right|}^{2}\delta \cdot \left[E_{C}\left(K\right)-E_{V}\left(K\right)-\hbar \omega \right]\right\}$$

where *ω* is angular frequency, *ε*_0_ is permittivity of vacuum, *e* and *m* are the charge and mass of electron, *BZ* represents the first Brillouin zone, *V* and *C* represent the valence and conduction bands, *E_V_*(*K*) and *E_C_*(*K*), respectively, denote the valence and conduction band intrinsic levels, *K* denotes the electron wave vector, *M_V,C_* is the transfer matrix and *a* represents the unit vector potential. 

The refractivity and extinction coefficients can be described as follows:

(9) $$n(\omega )=\frac{{\left[{\left(\varepsilon_{1}^{2}+\varepsilon_{2}^{2}\right)}^{1/2}+\varepsilon_{1}\right]}^{1/2}}{\sqrt{2}},\;\;\;\;k(\omega )=\frac{{\left[{\left(\varepsilon_{1}^{2}+\varepsilon_{2}^{2}\right)}^{1/2}-\varepsilon_{1}\right]}^{1/2}}{\sqrt{2}}$$

Then, the absorption coefficient and reflectivity are further deduced as follows:

(10) $$\alpha =\frac{4\pi k}{\lambda_{0}}$$

(11) $$R(\omega )=\frac{{\left(n-1\right)}^{2}+k^{2}}{{\left(n+1\right)}^{2}+k^{2}}$$

**Figure 5:**
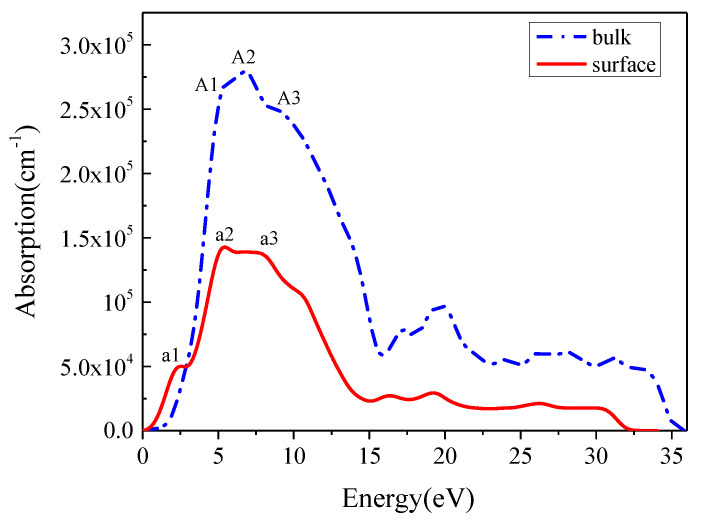
Absorption curves of In_0.87_Ga_0.13_As_0.25_P_0.75_ bulk and In_0.87_Ga_0.13_As_0.25_P_0.75_(001)β_2_(2×4) surface.

Optical absorption occurs when the light intensity attenuates with the penetration depth, and the absorption coefficient reflects the optical absorption intensity. The absorption coefficient is not only related to the material, but also varies with the light wavelength. For the large absorption coefficient, the light absorption is actually concentrated in the crystal surface layer. Figure 5 shows the absorption coefficients of In_0.87_Ga_0.13_As_0.25_P_0.75_ bulk and reconstruction surface.

From Figure 5, it can be found that the absorption peaks of In_0.87_Ga_0.13_As_0.25_P_0.75_(001) β_2_(2×4) surface largely attenuate compared to those of bulk In_0.87_Ga_0.13_As_0.25_P_0.75_ in almost the entire energy range, except that the surface absorption peak caused by the electronic transition of As 4p and P 3p states is higher than bulk absorption coefficient in the range of 0–2.86 eV. Bulk In_0.87_Ga_0.13_As_0.25_P_0.75_ has three absorption peaks A1, A2 and A3 that are, respectively, located at 5.24, 6.82 and 9.33 eV. Corresponding to the bulk, the surface also has three peaks a1, a2 and a3 that are, respectively, located at 2.14, 5.37 and 7.94 eV. Among these peaks, the absorption coefficients of A2 and a2 are 279,268 and 142,733 cm^−1^, which are the highest peaks for bulk and surface, respectively. From bulk to surface, the absorption peaks undergo redshift and peak values decrease. However, the surface has a higher absorption coefficient than the bulk in the low energy range of 0–2.901 eV, which proves that the reconstruction surface can increase the long-wave absorption.

**Figure 6:**
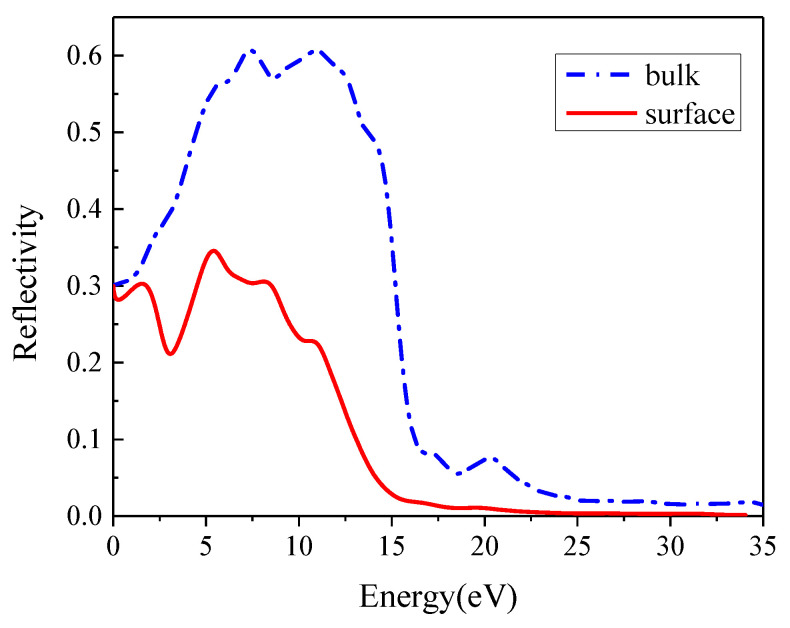
Reflection spectrum of In_0.87_Ga_0.13_As_0.25_P_0.75_ bulk and surface.

Figure 6 shows the calculated reflectivity of the bulk and In_0.87_Ga_0.13_As_0.25_P_0.75_(001)β_2_(2×4) reconstruction surface. Bulk In_0.87_Ga_0.13_As_0.25_P_0.75_ exhibits metal reflection characteristics in the range of 3.2–14.86 eV. Compared to the bulk, the surface reflectivity considerably decreases, and its high-reflection range becomes narrower as the falling edge redshifts. The considerable decrease in the surface reflectivity and absorption coefficient improves the surface transmissivity, which is conducive for the photons passing through the surface into the bulk, and more photoelectrons are excited. 

**Figure 7:**
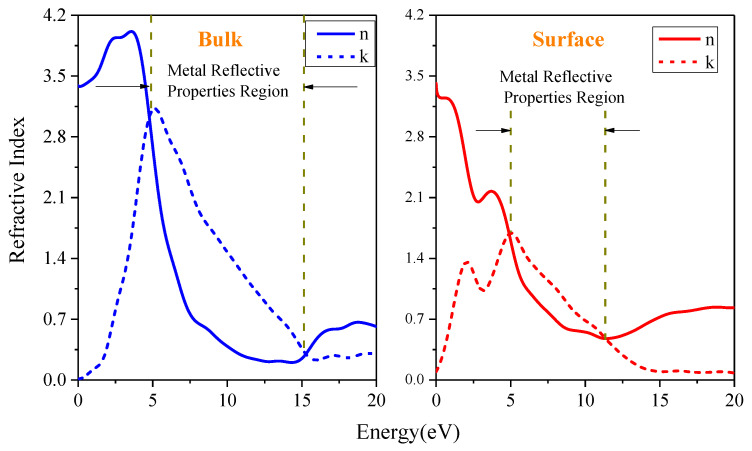
Complex refractive index of the In_0.87_Ga_0.13_As_0.25_P_0.75_ bulk and surface.

The complex refractive index curves are shown in Figure 7. The compositions of In_0.87_Ga_0.13_As_0.25_P_0.75_ are similar to In_0.89_Ga_0.11_As_0.24_P_0.76_. Near 1.25 eV, we calculated that the refractive index value of In_0.87_Ga_0.13_As_0.25_P_0.75_ is approximately 3.5, which is close to the refractive index range 3.47–3.5 of In_0.89_Ga_0.11_As_0.24_P_0.76_ obtained by Seifert and Runge [29]. Due to *k* > *n* and *ε*_1_ < 0 being in the ranges of 4.71–15.16 eV and 4.91–11.32 eV, respectively, for the In_0.87_Ga_0.13_As_0.25_P_0.75_ bulk and surface, they exhibit very strong reflection properties. This is consistent with the reflection spectrum. Here, we can also find that the high-reflection range of the surfaces becomes narrower compared to the bulk.

**Figure 8:**
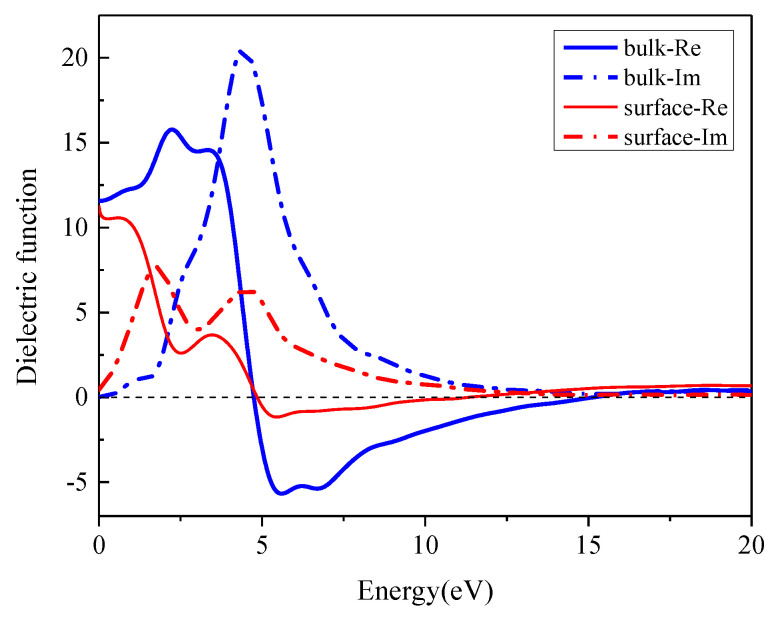
Dielectric function of the In_0.87_Ga_0.13_As_0.25_P_0.75_ bulk and surface.

Figure 8 shows the imaginary and real parts of the dielectric function of In_0.87_Ga_0.13_As_0.25_P_0.75_ surface and bulk. Compared with the bulk, the dielectric function of In_0.87_Ga_0.13_As_0.25_P_0.75_(001)β_2_(2×4) surface attenuates a lot. The real part *ε*_1_ appears negative peaks in the range of 5–10 eV, corresponding to the strong reflection regions of the In_0.87_Ga_0.13_As_0.25_P_0.75_ surface and bulk. In this energy range, the metal reflection characteristics of the bulk make it difficult for photons to propagate in it. As for the peaks of *ε*_2_, they are consistent with those of the absorption curve, and the surface peak value in the low-energy side is higher than that of the bulk. For surface, the dielectric function peaks undergo redshift and the peak values decrease.

## 4. Conclusions

First-principles methods are adopted to calculate the electronic structure, work function, formation energy, Mulliken population and optical properties of In_0.87_Ga_0.13_As_0.25_P_0.75_(001)β_2_(2×4) reconstruction surface. Results show that In_0.87_Ga_0.13_As_0.25_P_0.75_(001)β_2_(2×4) surface has minus formation energy and lower work function than GaAs(001)β_2_(2×4) surface, demonstrating that In_0.87_Ga_0.13_As_0.25_P_0.75_ reconstruction surface is stable and more conducive to the escape of low-energy photoelectrons. Compared to the bulk, the narrower bandgap and emerging energy levels of the reconstruction surface make electron transition easier. Under the action of dipole moment, the electrons transfer from the inner layers to the surface during the surface formation process. The optical properties between the surface and bulk are very different. By contrast, the absorption peaks of the surface undergo redshift and the peak values decrease. However, the surface has a higher absorption coefficient than that of the bulk in the low energy range of 0–2.901 eV, which proves that the reconstruction surface can increase the long-wave absorption. The surface reflectivity decreases a lot, and its high-reflection range becomes narrow as the falling edge redshifts. The strong decrease in the surface reflectivity and absorption coefficient improves the surface transmissivity, which is conducive to the photons passing through the surface into the bulk and exciting more photoelectrons. The dielectric function peaks of the surface move toward the lower energy region and the peak values decrease.

## Figures and Tables

**Table 1:** Variation of integral partial DOS of the surface relative to the bulk.

Variation	**In**			**Ga**			**As**		**P**	
	s	p	d	s	p	d	s	p	s	p
	−33%	−23.1%	−17%	−28%	−25.3%	−14.9%	−13%	−11.5%	+39.3%	−32.7%

**Table 2:** Mean Mulliken charge distribution in each layer of In_0.87_Ga_0.13_As_0.25_P_0.75_(001)β_2_(2×4) surface.

	First Layer	Second Layer	Third Layer	Fourth Layer	Fifth Layer	Sixth Layer	Seventh Layer
In	/	0.42	/	0.57	/	0.46	/
Ga	/	0.42	/	0.71	/	0.45	/
As	−0.21	/	−0.17	/	0.11	/	/
P	/	/	−0.55	/	−0.53	/	−0.31

## Data Availability

Not applicable.
